# Association of Air Pollution With Adiposity Rates in Active Runners and Inactive People

**DOI:** 10.1002/ajhb.70159

**Published:** 2025-10-28

**Authors:** Petr Kutac, Martina Dankova, Lukas Cipryan, Marek Buzga, Vitezslav Jirik, Vaclav Bunc, Martin Sigmund, Miroslav Krajcigr, Matthew Zimmermann, Daniel Jandacka

**Affiliations:** ^1^ Department of Human Movement Studies, Faculty of Education University of Ostrava Ostrava Czech Republic; ^2^ Institute for Research and Applications of Fuzzy Modeling University of Ostrava Ostrava Czech Republic; ^3^ Department of Physiology and Pathophysiology, Faculty of Medicine University of Ostrava Ostrava Czech Republic; ^4^ Department of Epidemiology and Public Health, Faculty of Medicine University of Ostrava Ostrava Czech Republic; ^5^ Centre for Epidemiological Research, Faculty of Medicine University of Ostrava Ostrava Czech Republic; ^6^ Faculty of Education Charles University Praha Czech Republic; ^7^ Application Centre BALUO, Faculty of Physical Culture Palacky University Olomouc Czech Republic; ^8^ Department of Sports Studies, Faculty of Education University of South Bohemia Ceske Budejovice Czech Republic; ^9^ School of Human Sciences The University of Western Australia Perth Australia

**Keywords:** air pollution, body fat, factors affecting adiposity, visceral fat

## Abstract

**Objectives:**

The aim of this cross‐sectional study is to investigate the association between long‐term air pollution (AP) exposure and adiposity, primarily visceral fat and secondary body fat in runners and inactive participants.

**Methods:**

This study included 945 individuals (male *n* = 505 and female *n* = 440). These included both active (runners: run ≥ 10 km/week) and inactive (did not follow the WHO 2020 PA recommendations) individuals. Dependent variables were body composition parameters fat mass index (FMI) and visceral fat (VFA), measured using dual‐emission X‐ray absorptiometry (DXA). A Hologic QDR (Horizon) bone densitometer was used for the measurement. Independent variables were AP values (PM_10_, PM_2.5_, NO_2_, BaP), for which lifetime exposure (LC_xp_) was calculated. Volume physical activity (PA), eating habits, and cardiorespiratory fitness (V̇O_2peak_) were analyzed as covariates.

**Results:**

The results showed that long‐term exposure to AP was not associated with increased adiposity (*p* > 0.05). However, age (*p* = 0.000), sex (*p* = 0.000), and V̇O_2peak_ (*p* = 0.000) were associated with VFA. Values for VFA increased with age, males had higher VFA than females, and VFA values decreased with increasing V̇O_2peak_ (*p* < 0.05) values. Furthermore, higher V̇O_2peak_ values were strongly associated with lower FMI (*p* = 0.000).

**Conclusions:**

Adiposity was not associated with AP in the studied population. Adiposity was affected mainly by lifestyle and associated cardiorespiratory fitness presented by V̇O_2peak_ values.

## 1 Introduction

Air pollution (AP) is a major problem, with more than 91% of the world's population living in areas where air pollution exceeds World Health Organization (WHO) limits (Raj 2020). According to WHO recommended air quality guidelines (AQG), annual levels of air pollutants should not exceed 5 μg/m^3^ (PM_2.5_), 15 μg/m^3^ (PM_10_), and 10 μg/m^3^ (NO_2_) (WHO 2021b). Furthermore, AP is associated with up to 7 million deaths per year, with these deaths likely due to the health effects associated with AP (mainly respiratory and cardiovascular diseases) (Bostan 2022; Lederer et al. 2021; Montone et al. 2023; Samet 2019; Tiotiu et al. 2020). Moreover, high levels of AP have shown to increase the incidence of neurodegenerative diseases (Wang et al. 2021). A person's lifestyle can also be negatively affected by AP, as individuals choose to limit their outdoor physical activity (PA) to mitigate the negative impact of AP on their health (An et al. 2018). However, this can lead to an increase in an individual's adiposity and an associated higher risk of several serious diseases (An et al. 2018).

Significant associations between AP and the prevalence of obesity in children and adults have been reported (Bowe, Gibson, et al. 2021; Furlong and Klimentidis 2020; Zhang et al. 2021). Obesity, as a chronic inflammation of adipose tissue, increases the risk of many chronic noninfectious diseases that are also associated with AP, including metabolic, oncological, and cardiovascular diseases, as well as musculoskeletal disorders (Kachur et al. 2017; Lin and Li 2021; Safaei et al. 2021). Additionally, these diseases lead to a reduction in quality of life and life expectancy. Worldwide, the prevalence of overweight and obesity increased by 27.5% in adults and 47.1% in children between 1980 and 2013 (Ng et al. 2014). Obesity rates increased for both sexes at all ages, regardless of geographic location, ethnicity, or socioeconomic status (Chooi et al. 2019). According to WHO data, in 2022, more than 1 billion people worldwide were living with obesity. Adult obesity rates have more than doubled since 1990 and quadrupled among children and adolescents aged 5–19. In the European Region, nearly 60% of adults and almost one in three children (29% of boys and 27% of girls) are overweight or living with obesity (WHO 2022). WHO reported in the adult population (18+ years) overweight in 39% of males, 40% of females and obesity in 11% of males and 15% of females (WHO 2021a). In children and adolescents (from 5 to 19 years) the prevalence of overweight is 19% in boys and 18% in girls and of obesity is 8% in boys and 6% in girls (WHO 2021a). According to Eurostat, 21.4% of males and 17.7% of females in the Czech Republic are obese (Eurostat 2022).

Obesity is defined as a disease process characterized by excessive body fat accumulation with multiple organ‐specific consequences' (Bray et al. 2017). One of the basic pathophysiological characteristics is the increased accumulation of body fat (Fernández‐Sánchez et al. 2011). However, there is no specified amount of body fat (BF) that is considered high—no standard for BF has been established. Currently, obesity is defined using BMI values or BMI percentile charts. The diagnosis of overweight and obesity using the body mass index (BMI), which cannot assess the fractionalization of body mass and therefore, the amount of body fat, is therefore inadequate (Gába and Přidalová 2014; Wong et al. 2021). Body fat percentage can also be used for diagnosing obesity; however, it does not account for body height. For this reason, some recent studies recommend using the fat mass index (FMI), which incorporates body height, distinguishes between fat and muscle mass, and is not influenced by fat‐free mass (Alpízar et al. 2020; Liu et al. 2013; Pereira‐da‐Silva et al. 2016). Human adipose tissue includes visceral adipose tissue, which is a key component in the onset and development of cardiovascular disease and type 2 diabetes (Jung et al. 2016; Korsić et al. 2011; Sokolov and Pisarevskaia 2007). The accumulation of visceral adipose tissue, leading to visceral or central obesity can lead to, among other things, insulin resistance, which increases the risk of the aforementioned diseases (Lubis et al. 2021).

The proportion of body fat is influenced by several exogenous factors, including age, sex (Gába and Přidalová 2014; Greendale et al. 2019), lifestyle and nutrition (Najjar and Feresin 2019), PA (Kutac et al. 2021), and socioeconomic status (Marmot and Bell 2019). Additionally, research has shown significant associations between AP and adiposity (Cai et al. 2022; Furlong and Klimentidis 2020). Thus, AP could be another important factor that can affect an individual's body mass and body composition. Air pollutants include chemical pollutants (e.g., NO_2_, SO_2_, CO_2_, O_3_, benzo[a]pyrene) or dust particles (PM_2.5–10_), for many of which there are legal emission limits. In the Czech Republic, the annual limit for NO_2_ is 40 μ/m^3^, benzene 5 μ/m^3^, PM_10_ 40 μ/m^3^, and PM_2.5_ 20 μ/m^3^ (Act on Air Protection Pub. L. No. Act n. 201/2012 Sb (2012); Czech Republic 2012). These are significantly higher values than those given in the recommended AQG WHO (WHO 2021b). Associations between AP and increases in body mass, BMI and body fat are likely due to changes in metabolism (Chen and Schwartz 2008; Han et al. 2022), adipose deposition (Heindel and vom Saal 2009; Tang‐Péronard et al. 2011), cravings and/or satiety, which is hormonally or neurologically regulated (Chen et al. 2019), and oxidative stress (Chuang et al. 2007). However, the results of recent research differ, with some studies finding no direct association between change in body mass and AP (James et al. 2017; White et al. 2016), whereas, others vary in the associations between different pollutants or health aspects. For example, a study of adults in the United States found levels of PM_2.5_ were associated with metabolic syndrome, not abdominal obesity (Wallwork et al. 2017). Furthermore, a study of a young population aged 3–17 years found levels of NO_2_, not PM_2.5_ or PM_10_, were associated with being overweight and obese (Bloemsma et al. 2019). Most recent studies have analyzed the association between AP and body mass or BMI; however, there is limited research addressing the association of AP with body fat or visceral fat.

Therefore, the aim of the current study was to investigate the association between long‐term air pollution exposure and body fat composition in both runners (run ≥ 10 km/week) and inactive individuals.

## 2 Materials and Methods

This cross‐sectional study is a part of the Healthy Aging in Industrial Environment Study‐Program 4 (4HAIE). The main objective of the 4HAIE study was to assess the effect of air pollution on health, wellbeing, PA, and aging. The study focused on the adult population in two regions of the Czech Republic. These are the Moravian–Silesian region (MSR) and the South Bohemian region (SBR). The MSR region contains heightened air pollution and has been a long‐term European hotspot of ambient benzo[a]pyrene and particulate matter pollutants (Hůnová 2020; Michalik et al. 2022). Exposure to benzo[a]pyrene AP in the MSR (Ostrava‐Radvanice) has been the highest in the European Union over the last decade (Sram et al. 2013). Conversely, the SBR is one of the least polluted regions in the Czech Republic with PM_2.5_ concentrations ranging from ≤ 10 to 12–17 (μg m^−3^) (System Air Quality Information 2018).

### 2.1 Participants

The 4HAIE study participants included 1314 individuals (706 males, 608 females), aged between 18 and 65 years, who met all the criteria for inclusion in the 4HAIE Study—Program 4 (CZ .02.1.01/0.0/0.0/16_019/0000798). Participants were recruited via a stratified recruitment strategy from March 1, 2019 to August 30, 2021, with the assistance of a professional marketing and social science research company in the MSR and SBR.

Common inclusion criteria included: being aged between 18 and 65 years; nonsmoking; able to perform normal PA including running (i.e., no medical restrictions on PA mandated by a physician); residing in the region for the past 5 years, without any plans to move in the following year; with internet access and using a smartphone (with iOS or Android 5.0 or higher).

The common exclusion criteria included: acute (in the past 6 weeks) medical issues (pain, injury, surgery) preventing normal PA, other acute illnesses; pregnancy; radiological examination in the past 7 days; factors that would exclude a graded exercise test or magnetic resonance examination (such as a pacemaker, radioactive or surgical devices/implants, insulin pump).

Inclusion criteria for runners included: running as a main exercise activity, > 150 min of moderate or > 75 min of strenuous PA per week (or an equivalent combination of moderate and strenuous PA) (WHO 2020), ≥ 10 km of running per week for at least 6 weeks prior to the tests, intending to continue running for the next 12 months, residing in the region for the past 5 years, without any plans to move in the following year, with internet access and using a smartphone (with iOS or Android 5.0 or higher). Total implemented exercise regime was usually higher (Kutac et al. 2024).

Inclusion criteria for “inactive” individuals included: < 150 min of moderate or < 75 min of strenuous PA per week (i.e., not meeting current public PA guidelines) (WHO 2020), capable of running, but not running or running irregularly < 6 weeks prior to testing, no contraindications to exercise, residing in the region for the past 5 years, without any plans to move in the following year, with internet access and using a smartphone (with iOS or Android 5.0 or higher).

Only individuals who met the inclusion criteria for one of the two groups (runners, inactive) were included in the study. Participants who did not meet the inclusion criteria for one of the two groups were excluded. Participants who met the WHO's PA volume requirements but were not runners were also excluded from the study.

The final number of participants in the present study was 945 (505 males, 440 females), with 369 participants being excluded due to missing data. Of these, 219 (16.7%) had missing data for lifetime exposure to AP, 112 (8.5%) had missing data for the graded exercise test (GXT), 28 (2.1%) from the runner's group had missing data on running, and 10 (0.8%) had missing data on eating habits. The baseline characteristics of the participants are presented in Table 1.

**TABLE 1:** Basic characteristics of the participants.

Parameters	MSR (*n* = 542)		SBR (*n* = 403)	
	Male (*n* = 284)	Female (*n* = 258)	Male (*n* = 220)	Female (*n* = 183)
	*M* ± SD	*M* ± SD	*M* ± SD	*M* ± SD
Age (year)	38.86 ± 11.78	39.11 ± 12.28	36.78 ± 11.71	37.34 ± 12.61
BH (cm)	180.43 ± 6.51	167.99 ± 6.08	181.05 ± 6.65	168.12 ± 6.34
BM (kg)	81.69 ± 11.24	66.89 ± 11.88	81.64 ± 12.48	67.24 ± 13.00
BF (kg)	21.87 ± 6.59	24.05 ± 8.13	21.63 ± 7.22	24.64 ± 8.69
FMI (kg/m^2^)	6.55 ± 1.99	8.35 ± 2.85	6.45 ± 2.21	8.47 ± 2.94
VFA (cm^2^)	95.84 ± 45.41	65.50 ± 41.46	89.27 ± 40.37	60.80 ± 39.02
V̇O_2peak_ (mL/kg/min)	45.98 ± 9.49	36.63 ± 8.69	46.69 ± 9.99	36.52 ± 8.63
PA (min/week)	479.43 ± 480.28	442.91 ± 505.70	513.51 ± 545.58	433.21 ± 443.51
Eating (WHO rec)	6.93 ± 2.31	6.11 ± 2.39	7.35 ± 2.42	6.26 ± 2.65
PM_10_ (μg/m^3^)	40.66 ± 8.55	40.22 ± 8.09	17.91 ± 3.85	18.34 ± 4.14
PM_2.5_ (μg/m^3^)	30.92 ± 6.74	30.52 ± 6.48	14.31 ± 2.95	14.61 ± 3.17
NO_2_ (μg/m^3^)	17.94 ± 5.56	17.35 ± 5.62	10.17 ± 2.81	10.48 ± 3.04
BaP (ng/m^3^)	3.50 ± 2.13	3.47 ± 2.11	0.39 ± 0.04	0.39 ± 0.04

Abbreviations: BaP, benzo[a]pyrene; BF (kg), body fat; BH, body height; BM, body mass; Eating (WHO rec), eating WHO dietary recommendation; FMI, fat mass index; *M*, mean; MSR, the Moravian–Silesian region; *n*, frequency; PA, physical activity; SBR, South Bohemian region; SD, standard deviation; VFA, visceral fat; V̇O_2peak_, maximal oxygen consumption.

### 2.2 Procedures

Participation in the study was voluntary, and all the participants gave written informed consent prior to the initial measurements in the laboratory. Details about the 4HAIE study design, methods, and measurement protocol are available in previous publications including: the physiological and anthropometric protocol (Cipryan et al. 2020); the behavioral, psychological, and neuroimaging protocol (Elavsky et al. 2021); and the biomechanical and musculoskeletal protocol (Jandacka et al. 2020). The study was approved by the Ethics Committee of the University of Ostrava (protocol code OU‐87674/90‐2018 and date of approval November 29, 2018) and followed the principles of the Helsinki Declaration.

### 2.3 Somatic Measurements

To ensure standard conditions of measurement, the participants were admitted to the research center 15 h prior to measurement (they slept in the sleep laboratory). Measurement commenced at 8:30 a.m. the next morning. The participants were measured barefoot, in their underwear. All the measurements were taken in the following order: body height (BH), body mass (BM), body composition. BH and BM, which are the input parameters for the bone densitometer software, were measured using the InBody BSM 370 stadiometer (Biospace, South Korea). The analysis of body composition included body fat (BF) and visceral fat (VFA) expressed by area (cm^2^). For the analysis of weight status, instead of body fat analysis we used the FMI: BF (kg)/BH (m^2^). To measure body composition, the DXA method was used, with a Hologic QDR Horizon A bone densitometer (Hologic, Waltham, MA, USA). The positions during the measurement and the measured segments are presented in the protocol article (Cipryan et al. 2020). The bone densitometer used has a typical error of measurement (TEM) for body fat (BF kg), depending on sex, in the range of 0.25–0.52 kg (Kutáč et al. 2019). The densitometer is regularly calibrated and undergoes technical safety control (TSC). Calibration is regularly performed by an authorized company that handles the installation, control measurements, calibration, and servicing of the device. Additionally, at the beginning of each measurement day, a daily quality control (DQC) is performed to check whether the currently measured values on the spinal phantom (area BMC and the calculated BMD) are within the allowed tolerance from the calibration scans performed by the authorized Czech company.

### 2.4 Physical Activity Assessment

Results from the ACLS questionnaire were used to measure volume PA (Kohl et al. 1988). The questionnaire was administered online via the Qualtrics^XM^ platform (Qualtrics, Provo, UT, USA) along with other baseline measures. The questionnaire contains the assessment of 14 different physical activities. Before completing the questionnaire, participants were instructed to indicate which of the following activities they had done regularly during the past month. We analyzed the number of episodes per week and the duration of each episode for each PA recorded. Based on these values, we calculated the duration of each type of PA per week. Total volume PA is given by the sum of all types of PA per week.

### 2.5 Eating Habits

Questions from the baseline questionnaire, completed online using Qualtrics^XM^, were used to analyze eating habits. The baseline questionnaire included questions on demographic data, socioeconomic status, risk perception, health status, lifestyle, and quality of life. Questions assessing eating habits were part of a group of questions assessing lifestyle. These questions were included in the baseline questionnaire from the Health, Lifestyle and Environment (HELEN) study, which assesses the health status of the population in the country (The National Institute of Public Health (NIPH) 2003; Žejglicová et al. 2016). For the item Eating (WHO dietary recommendations), diet was scored (min. 0, max. 16 points), with a higher score indicating fewer WHO dietary recommendations are followed. For the item Eating (healthy/unhealthy), diet was scored 0 or 1 (0 = *relatively healthy diet*; healthy eating principles are not followed for less than 10 food groups, 1 = *unhealthy diet*; healthy eating principles are not followed for more than 10 food groups). Before completing the questionnaire, participants were instructed to indicate how often on average they consumed the listed foods.

### 2.6 Graded Exercise Test (GXT)

Participants performed a laboratory GXT on a motorized treadmill (Rodby RL 2500E) to determine maximum aerobic power (V̇O_2peak_). Prior to the GXT, participants completed 3 min of walking at 5.0 km/h to familiarize with the treadmill. The GXT protocol then commenced at 6.0 km/h (1% inclination), with speed subsequently increasing by 1.0 km/h every minute until volitional exhaustion. Expired air was continuously monitored to analyze O_2_ and CO_2_ concentrations during the GXT with a breath‐by‐breath system (Blue Cherry, Geratherm Medical AG, Germany). The highest average O_2_ consumption measured over a 30s period was used to determine V̇O_2peak_. All sessions were conducted in the afternoon, at least 3 h after the participants' last meal and in a thermally controlled laboratory (21°C, 40% relative humidity). Each participant was advised not to participate in any vigorous activity 24 h prior to the test. Prior to completing the GXT, if participants did not pass the Physical Activity Readiness Questionnaire, they were not allowed to perform the GXT unless explicit permission (given by a medical doctor) was provided. Blood pressure (BP) was also checked before the GXT. If BP values were ≥ 150/90 mmHg, the participant was not allowed to perform GXT, but continued in the study protocol. The detailed protocol of the GXT has been published elsewhere (Cipryan et al. 2020).

### 2.7 Air Pollution—Calculating Lifetime Exposure

The lifetime exposure calculation methodology, utilizing historical time series analysis, was developed and published by Machaczka et al. (2023), building upon the district‐level air pollutant concentration analysis method originally proposed by Michalik et al. (2022). This study employed time series data from PM_10_, PM_2.5_, NO_2_, benzene, and benzo(a)pyrene, sourced from national databases, for model calculations. Annual average concentrations were derived by integrating: measured and mathematically modeled air pollution data from the Czech Hydrometeorological Institute (CHI n.d.) for the years 1997–2017, custom spatial and temporal data modeling based on correlation and regression analysis of measured data, emission balance data and dust fallout data for the years 1960–1997, and correlation analysis of documented industrial production data, a key factor in pollutant emissions. For districts lacking specific industrial production data, a constant trend extrapolation was used to extend the time series prior to 1960.

Lifetime exposure to air pollutants was quantified as the lifetime average exposure concentration (LC_xp_) of each individual air pollutant (*x*) and proband (*p*). This calculation utilized the average concentration derived from historical time series of air pollutant concentrations, as previously described. Specifically, the concentration *IC*_*dn*_ was calculated for pollutant *c* in district *d*, corresponding to the years *y* that the proband resided in that region. From these annual concentrations, lifetime exposures were determined using the following calculation:

$${\mathrm{LC}}_{\mathrm{xp}}=\frac{\sum_{\;i=y}^{\;i=y+a}\left(c_{\mathrm{xdi}}\right)}{a}$$

where (LC_xp_) represents the lifetime exposure of participant (*p*) to pollutant (*c*), and (*c*_*dn*_) is the annual average pollutant concentration (*x*) in district (*d*), where the participant resided in year (*i*). The annual concentrations from the participant's birth year (*y*) to their current age (*a*) are summed and then divided by their age to obtain the lifetime average.

### 2.8 Statistical Analysis

To explain the relationship between independent variables—specifically, PM_10_, PM_2.5_, NO_2_, benzo[a]pyrene, and location—and dependent variables, which include whole body fat, which was subsequently used for the analysis of the FMI and visceral fat (VFA), a linear regression model was employed. Covariates of age, sex, education level, dietary habits (“eating” rated as points on a scale from healthy to unhealthy; and the binary “eating” encoding healthy/unhealthy dietary habits), weekly volume PA (minutes per week), and V̇O_2peak_ were incorporated into the model.

To meet the basic assumptions for the correct application of the linear regression model, we had to remove from the set of explanatory variables those that were correlated with each other (see Table A2). From this, only the variable PM_10_ was retained from the set of independent variables. This decision was made because the variable PM_10_ is continuous and has been shown to effectively capture the level of pollution in a specific individual's environment.

The preprocessing of the data involved removing entries with missing arguments. Subsequently, the best normalize method (Peterson 2021; Peterson and Cavanaugh 2020) was applied to transform the non‐categorical data. It is an adaptive transformation method that employs a data‐driven approach to select the optimal normalization method. It evaluates several transformations, including Box‐Cox, Yeo‐Johnson, ordered quantile normalization, arcsinh, and log transformations, and chooses the one that yields the distribution closest to normality, as determined by the Pearson *P* statistic. The automated selection criterion prioritizes the transformation that minimizes deviation from normality, thereby enhancing the suitability of the transformed variable for statistical modeling assumptions (Table 2).

**TABLE 2:** The values of Pearson's correlations between calculated lifetime (LC_xp_) exposure to different pollutants.

	Location	PM_10_ (μg/m^3^)	PM_2.5_ (μg/m^3^)	NO_2_ (μg/m^3^)	BaP (ng/m^3^)
Location	1.00	0.79	0.78	0.64	0.79
PM_10_ (μg/m^3^)	0.79	1.00	0.98	0.88	0.89
PM_2.5_ (μg/m^3^)	0.78	0.98	1.00	0.87	0.92
NO_2_ (μg/m^3^)	0.64	0.88	0.87	1.00	0.84
BaP (ng/m^3^)	0.79	0.89	0.95	0.84	1.00

Abbreviation: BaP, benzo[a]pyrene.

## 3 Results

**FIGURE 1:**
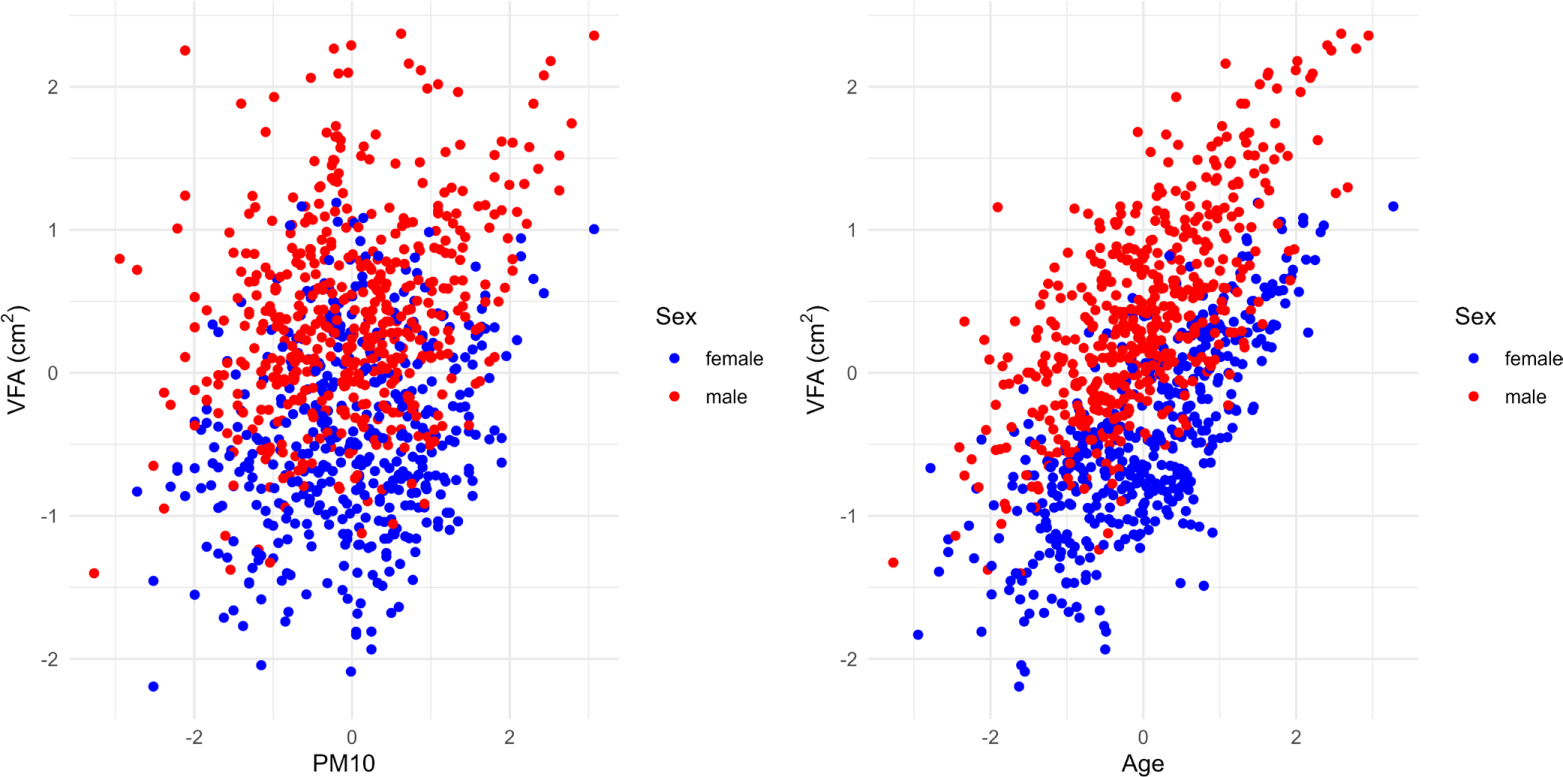
Visualizations of the resulting models for visceral fat area (VFA) with respect to (PM_10_, sex) and (age, sex), respectively, where all variables are transformed.

**FIGURE 2:**
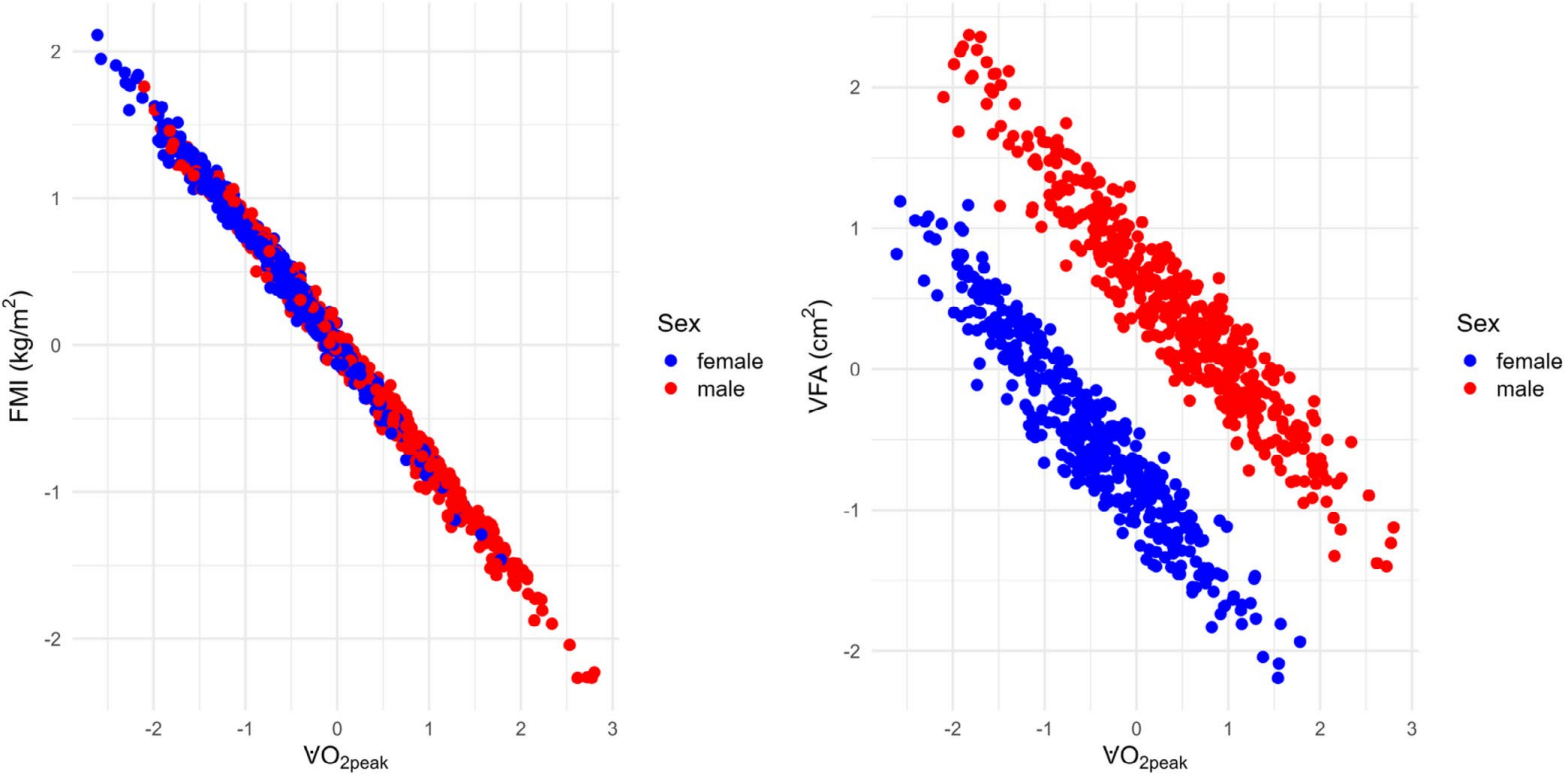
Visualization of the resulting models for the fat mass index and visceral fat with respect to V̇O_2peak_ and Sex, where all variables are transformed.

**FIGURE 3:**
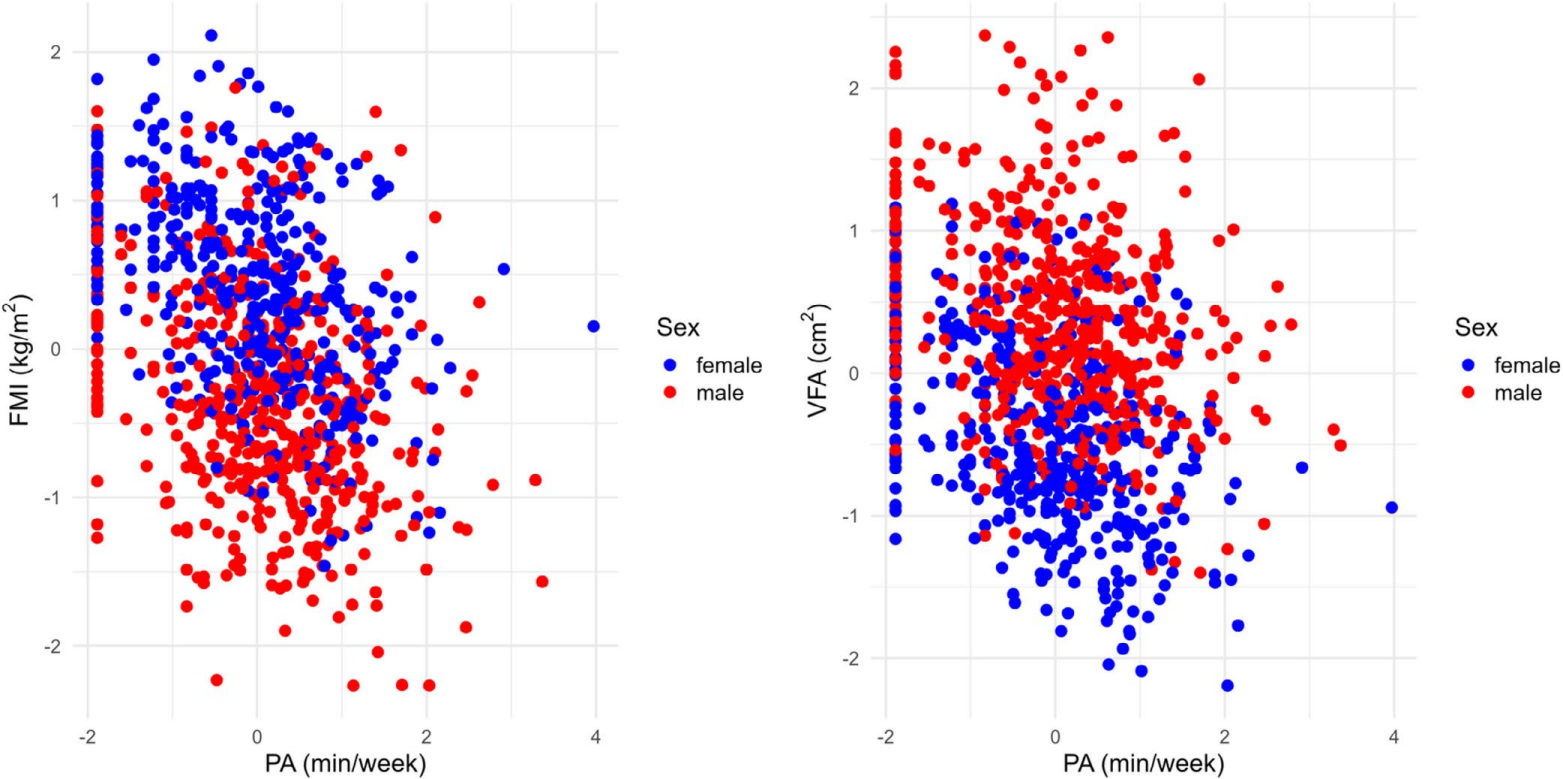
Visualizations of the resulting models for fat mass index and visceral fat with respect to physical activity and Sex, where all variables are transformed.

The input values, mean and standard deviation, median and quartile deviation of the monitored somatic parameters, pollutants, and covariates for the linear regression models are summarized for both the original and the transformed values in Table 3. Additionally, the statistical analysis results are presented in Table 4. Some selected visualizations of the resulting models are presented in Figures 1, 2, 3.

**TABLE 3:** Input values dependent variables (somatic parameters), independent variables (lifetime (LC_xp_) exposure to pollutants) and covariates for linear regression models.

Parameters	Values before transformation		Values for the transformation	
	Me ± QD	*M* ± SD	Me ± QD	*M* ± SD
Somatic parameters (dependent variables)				
FMI (kg/m^2^)	6.75 ± 3.21	7.39 ± 2.66	−0.04 ± 1.33	0.00 ± 1.00
VFA (cm^2^)	68.43 ± 24.47	79.38 ± 44.58	0.00 ± 0.67	0.00 ± 1.00
Lifetime (LC_xp_) exposure to pollutants (independent variables)				
Location	2.00 ± 0.50	1.57 ± 0.49		
PM_10_ (μg/m^3^)	30.91 ± 11.55	30.94 ± 13.02	0.00 ± 0.68	0.00 ± 1.00
PM_2.5_ (μg/m^3^)	23.33 ± 8.37	23.80 ± 9.71	0.00 ± 0.68	0.00 ± 1.00
NO_2_ (μg/m^3^)	12.76 ± 4.76	14.54 ± 5.90	−0.01 ± 0.67	0.00 ± 1.00
BaP (ng/m^3^)	1.23 ± 1.28	2.17 ± 2.22	0.00 ± 0.67	0.00 ± 1.00
Covariates				
Sex	2.00 ± 0.50	1.54 ± 0.50		
Age (year)	39.22 ± 9.17	38.14 ± 11.94	0.00 ± 0.67	0.00 ± 1.00
Education	3.00 ± 1.00	3.78 ± 1.20		
Eating (WHO rec)	7.00 ± 1.50	6.68 ± 2.48	0.09 ± 0.57	0.00 ± 0.98
Eating (h/unh)	1.00 ± 0.00	1.13 ± 0.33		
PA (min/week)	352.50 ± 217.50	440.99 ± 392.77	0.04 ± 0.64	0.00 ± 1.00
V̇O_2peak_ (mL/kg/min)	41.90 ± 17.50	41.79 ± 10.43	0.01 ± 0.72	0.00 ± 1.00

Abbreviations: BaP, benzo[a]pyrene; Eating (h/unh), healthy/unhealthy; Eating (WHO rec), eating WHO dietary recommendation; FMI, fat mass index; *M*, mean; Me, median; PA, physical activity; QD, quartile deviation; SD, standard deviation; VFA, visceral fat; V̇O_2peak_, maximal oxygen consumption.

**TABLE 4:** Linear regression models of visceral fat (VFA) and whole body fat (BF_WB_).

Parameters	Estimate	(95% CI)	Std. error	*p*
VFA (cm^2^): *R* ^2^ = 0.643				
Age (year)	0.230	(0.181, 0.279)	0.025	0.000***
Sex2	1.404	(1.309, 1.499)	0.048	0.000***
PM_10_ (μg/m^3^)	0.038	(−0.003, 0.079)	0.021	0.067^NS^
Education2	−0.129	(−0.262, 0.003)	0.068	0.056^NS^
Education3	−0.068	(−0.201, 0.065)	0.068	0.315^NS^
Eating (WHO rec)	0.039	(−0.0014, 0.093)	0.027	0.151^NS^
Eating (h/unh)	−0.08	(−0.235, 0.064)	0.076	0.261^NS^
PA (min/week)	−0.027	(−0.070, 0.016)	0.022	0.213^NS^
V̇O_2peak_ (mL/kg/min)	−0.587	(−0.642, −0.533)	0.028	0.000***
FMI (kg/m^2^): *R* ^2^ = 0.644				
Age (year)	−0.044	(−0.092, 0.005)	0.025	0.079^NS^
Sex2	0.045	(−0.050, 0.139)	0.048	0.353^NS^
PM_10_ (μg/m^3^)	−0.012	(−0.052, 0.029)	0.021	0.575^NS^
Education2	0.111	(−0.022, 0.243)	0.067	0.102^NS^
Education3	0.145	(0.013, 0.278)	0.067	0.032*
Eating (WHO rec)	0.016	(−0.038, 0.070)	0.027	0.558^NS^
Eating (h/unh)	−0.136	(−0.285, 0.014)	0.076	0.075^NS^
PA (min/week)	−0.035	(−0.078, 0.008)	0.022	0.108^NS^
V̇O_2peak_ (mL/kg/min)	−0.819	(−0.873, −0.765)	0.028	0.000***

Abbreviations: 95% CI, confidence interval; Eating (h/unh), healthy/unhealthy; Eating (WHO rec), eating WHO dietary recommendation; Education2, 2 secondary school diploma, Education3, higher vocational, university and higher; PA, physical activity; *R* ^2^, *R* squared (coefficient of determination); Sex2, male; V̇O_2peak_, maximal oxygen consumption.

***p < 0.001, **p < 0.01, *p < 0.05, ^NS^ *p* > 0.05—not significant.

In Table 3, categorical data were not transformed. These categories are location (1 = *unpolluted area*—SBR, 2 = *polluted area*—MSR), sex (1 = *female*, 2 = *male*), education (1 = *basic* (even unfinished), *apprentice, secondary vocational without GCSE*, 2 = *secondary school diploma* (general and vocational), 3 = *higher vocational* (post‐secondary, DiS); *university and higher* (Bc., MSr., Ing., Ph.D.)), eating healthy/unhealthy (1 = *healthy*, 2 = *unhealthy*).

PM10 was not associated with visceral fat or FMI (*p* > 0.05). Visceral fat (VFA) was associated with age, sex, and cardiorespiratory fitness (V̇O_2peak_) (*p* < 0.05). FMI was associated with education (Education3) and with cardiorespiratory fitness (V̇O_2peak_) (*p* < 0.05).

The correlation analysis shows (see Table A1) that VFA (cm^2^) is not meaningfully related to environmental exposures, eating habits, education levels, and PA. More relevant are sex and age showing moderate positive correlations (0.43 and 0.48, respectively), while a moderate negative correlation is observed with V̇O_2peak_ (−0.37), indicating that better cardiorespiratory fitness is linked to lower visceral fat. For FMI (kg/m^2^), correlations with environmental exposures and eating habits are negligible, sex and age show weak relations (−0.35 and 0.29, respectively), and PA is inversely related. The strongest finding is a very strong negative correlation with V̇O_2peak_ (−0.78), highlighting the close link between higher fitness and lower FMI.

In all subsequent figures, solid circles represent predicted values from the transformed input data. Colors differentiate between sexes.

In the graphs in Figure 1, the values of VFA for female (Sex1) with respect to either, age or PM_10_ are on average smaller although they are not clearly distinguishable. And obviously, values of VFA increase with age which is reflected by the coefficient of the regression model.

In Figure 2, the associations of FMI and VFA with V̇O_2peak_ are shown separately for each sex. With increasing V̇O_2peak_, both FMI and VFA decrease markedly, indicating that better cardiorespiratory fitness is strongly linked to lower fat accumulation. The negative relationship between FMI and V̇O_2peak_ appears almost identical in females and males, with no obvious sex difference. In contrast, for VFA, a clear separation by sex is observed: males have consistently higher visceral fat values compared to females across the entire range of V̇O_2peak_.

In Figure 3, the associations of FMI and VFA with physical activity (PA, min/week) are shown for both sexes. In contrast to the previous case with V̇O_2peak_, no clear sex‐related differences are visible for either FMI or VFA. In both plots, a clustering of data points is evident at the lowest PA values, indicating that many participants reported very limited or no PA. Beyond this, the scatter is wide, suggesting considerable interindividual variability. Although higher PA tends to be weakly associated with lower FMI and VFA, the relationship is not strong and shows substantial variation across individuals.

## 4 Discussion

The aim of the study was to investigate the association between long‐term AP exposure versus adiposity, primarily visceral fat and secondary body fat in runners and inactive participants. Current values are not essential for the effect of AP on the body, but the long‐term burden caused by air pollution (Stafoggia et al. 2022). The current study found that long‐term exposure to AP, represented by lifetime exposure to PM_2.5_, PM_10_, NO_2_, BaP, was not associated with increased FMI or VFA, regardless of the location in which the participant resided. However, both FMI and VFA were associated with age, sex, education, and V̇O_2peak_ (Table 4). It can be assumed that runners have a higher volume of PA than inactive participants, which is related to their higher VO_2peak_ values. The results indicate that adiposity (FMI, VFA) is significantly associated with VO_2peak_, and this is the reason for lower FMI and VFA values in runners compared to inactive participants. Unfortunately, there are currently no relevant minimum lifetime AP exposure levels that are considered pathological. All available standards and recommendations, only give short‐term limits for AP (annual at most).

Monitoring the effect of AP is essential for adiposity, as elevated concentrations of air pollutants can result in metabolic dysfunction due to increased oxidative stress, which can lead to inflammation of adipose tissue (Xu et al. 2011), lipid accumulation in the liver (Liu et al. 2014), and reduced glucose utilization in skeletal muscle (Liu et al. 2014). Thus, AP can indirectly affect body mass. Some studies have shown associations between elevated BF and elevated PM_2.5_ and PM_10_ (Bowe, Xie, et al. 2021; Cai et al. 2022; Furlong and Klimentidis 2020; Liu et al. 2020). However, several studies have not shown these associations. The likely cause is the absence of long‐term AP data and different measurement methodology. While Deschenes et al. (Deschenes et al. 2020) showed an association between PM_2.5_ and obesity prevalence based on analyses in China, they also pointed to the significant influence of socioeconomic factors such as education and living in a high‐ or low‐poverty neighborhood. Additionally, the results of a meta‐analysis that analyzed 66 studies that looked at associations between AP, body mass and obesity were inconsistent (inconclusive). It showed that 44% of these studies were positively associated with body mass, 44% of studies showed no association, and in 12% of studies BM was negatively associated with AP (An et al. 2018). The results of a second meta‐analysis that looked at the association of AP with increased body mass did find positive associations between BM and AP, but these were from developing countries (Huang et al. 2020). In developed regions of Europe and North America, studies have reported only nonsignificant weight changes associated with AP. The reason for these weak associations is probably due to the reduction of pollution in these developed countries (Huang et al. 2020), but also that an active lifestyle and diet may reduce the negative effects of AP on the development of obesity. This may also account for our findings. This seems to be confirmed by the results of another study on the 4HAIE cohort, which showed no association between long‐term air pollution versus biomarkers of oxidative status and inflammation in the Czech Republic population of 18–65 years old runners and nonrunners (Cipryan et al. 2024).

### 4.1 Factors Significantly Associating With Visceral Fat (VFA)

VFA values were significantly associated with sex, age, and V̇O_2peak_ (Table 4). The model shows that males in our study had higher VFA values than females (Figures 1, 2, 3), which is consistent with the results of other studies that have examined sex variation in VFA (Demerath et al. 2007; Panchu et al. 2019). Moreover, our finding of a positive association between increasing VFA values and increasing age in both sexes is consistent with results in several recent studies that have examined populations of both sexes, normal weight, and obese individuals (Higgins et al. 2022; Kutac et al. 2023; Panchu et al. 2019). The negative association we found between V̇O_2peak_ and VFA has also been demonstrated in several studies. Whether it was nonathlete adolescents (male, female), young soccer players (male), or obese women during a weight loss program, higher V̇O_2max_ values were always associated with lower VFA values (Agustiyawan 2022; Parikh et al. 2017; Wee et al. 2004).

### 4.2 Factors Significantly Associated With the Fat Mass Index (FMI)

Values for FMI (kg/m^2^) were primarily dependent on V̇O_2peak_, with additional contributions from education level (Education3) and, to a lesser extent, age, dietary balance, and PA (Table 4).

The significant association between FMI and V̇O_2peak_ is related to the PA (running) performed by the runners in our study. PA is one of the key factors influencing V̇O_2peak_ values (Meyler et al. 2021). However, the overall volume of performed PA is not related to FMI. Oxygen intake is primarily determined by the intensity of the performed physical load (Astrand et al. 2003). Therefore, the total volume of physical load performed is a necessary but not sufficient predictor of changes in FMI. Numerous studies in both children and adults confirm the positive effect of PA intensity on body fat reduction (Bailey et al. 2007; Mark and Janssen 2011; Schwendinger et al. 2025). PA intensity is reflected specifically by V̇O_2peak_ (Roosz et al. 2025).

The positive association between Education3 (highest education) and FMI in our study did not support the negative relationship between education, BMI values, and BF presented in many studies (Kim 2016; Pikhart et al. 2007; Jusof and Ramli 2019). The main factors associated with lower BF levels and lower risk of obesity include lifestyle, socioeconomic disadvantage, and mental health (Bartoskova Polcrova et al. 2024). Although these factors are associated with lower levels of education, this association may not be evident in some population groups. Even individuals with less education may have access to relevant information and, as a result, the motivation to adopt a healthy lifestyle. This seemed to be the case for our probands as well, as motivated individuals across education levels were more likely to report to our project and did not differ in factors influencing BF (FMI) values depending on educational attainment.

## 5 Strengths and Limitations

The main strengths of our study are that it includes a large cohort across multiple age groups, and AP was assessed based on lifetime exposure. Multiple factors that are known to influence body composition, such as sex, AP, eating habits, cardiorespiratory fitness, and socioeconomic status (education), were also accounted for in the linear regression model used.

This study also has some limitations. The first limitation is selection bias, as participants needed to meet the selection criteria of the 4HAIE study (location, age, sex, and PA status) (Cipryan et al. 2020). The second limitation is the self‐assessment of PA and eating habits, which are subject to various forms of bias, although standardized assessment tools were used. Third, the study cohort is skewed toward participants with a higher education level; therefore all education levels were not evenly distributed. Education level was not included among the common inclusion criteria in the 4HAIE project. Fourth, we were not able to show causality of the investigated dependent and nondependent variables due to the specific cross‐sectional study design. We can speculate that the difference in AP exposure between those two regions (SBR and MSR) in the Czech Republic is not sufficient to be statistically detected.

## 6 Conclusions

We demonstrated that AP is not associated with adiposity in the studied population of active runners and nonrunners. Adiposity was mainly affected by intensity PA (cardiorespiratory fitness). It is a component of lifestyle habits. Thus, individuals can very actively influence their adiposity through an active lifestyle, even when exposed to AP. This is not to diminish the importance of AP and its effect on body fat and visceral fat (or adiposity).

## Author Contributions

**Petr Kutac:** conceptualized and designed this study, designed the data collection instruments, collected data, carried out the initial analysis, drafted the initial manuscript, and reviewed and revised the manuscript. **Martina Dankova:** data analysis, statistical programming, regression modeling, visualization, writing – review and editing. **Lukas Cipryan, Marek Buzga, Vaclav Bunc, Matthew Zimmermann, and Daniel Jandacka:** conceptualized and designed this study, reviewed and revised the manuscript, and critically reviewed the manuscript for important intellectual content. **Vitezslav Jirik:** analyzed air pollution data, conceptualized and designed this study, reviewed and revised the manuscript, and critically reviewed the manuscript for important intellectual content. **Martin Sigmund:** designed the data collection instruments, collected data, carried out the initial analysis, and critically reviewed the manuscript for important intellectual content. **Miroslav Krajcigr:** analyzed the data, prepared tables, critically reviewed the manuscript for important intellectual content, and approved the final draft.

## Ethics Statement

The study was approved by the Ethics Committee of the University of Ostrava (protocol code: OU‐87674/90‐2018 and date of approval: November 29, 2018; protocol code: OU‐42093/90‐2024 and date of approval: April 24, 2024) and followed the principles of the Helsinki Declaration.

## Consent

The authors have nothing to report.

## Conflicts of Interest

The authors declare no conflicts of interest.

## Data Availability

The data that support the findings of this study are available from the corresponding author upon reasonable request.

## Appendix A

**TABLE A1:** Correlations between the independent variables with covariates and the dependent variables.

Row_names	FMI (kg/m^2^)	VFA (cm^2^)
Location	0.00	0.06
(LC_xp_) PM_10_ (μg/m^3^)	0.06	0.19
(LC_xp_) PM_2.5_ (μg/m^3^)	0.06	0.18
(LC_xp_) NO_2_ (μg/m^3^)	0.08	0.18
(LC_xp_) BaP (ng/m^3^)	0.03	0.12
Sex	−0.35	0.43
Age (year)	0.29	0.48
PA (min/week)	−0.23	−0.20
Education	−0.00	0.01
Eating (WHO rec)	−0.03	0.08
Eating (h/unh)	−0.02	0.03
V̇O_2peak_(mL/kg/min)	−0.78	−0.37

Abbreviations: Eating (WHO rec), eating WHO dietary recommendation; LC_xp_, lifetime exposure.

**TABLE A2:** Correlations between the independent variables with covariates.

Row_names	Location	PM_10_ (LC_xp_)	PM_2.5_ (LC_xp_)	NO_2_ (LC_xp_)	BaP (LC_xp_)	Gender	Age	PA (min/week)	Education	Eating (WHO rec)	Eating (h/unh)	V̇O_2peak_ (mL/kg/min)
Location	1.00	0.79	0.78	0.64	0.79	−0.02	0.07	0.00	0.03	−0.06	−0.06	−0.03
(LC_xp_) PM_10_ (μg/m^3^)	0.79	1.00	0.98	0.88	0.89	−0.01	0.31	0.00	0.04	−0.10	−0.07	−0.09
(LC_xp_) PM_2.5_ (μg/m^3^)	0.78	0.98	1.00	0.87	0.92	0.00	0.31	0.00	0.07	−0.09	−0.09	−0.09
(LC_xp_) NO_2_ (μg/m^3^)	0.64	0.88	0.87	1.00	0.84	0.00	0.31	0.00	0.05	−0.07	−0.06	−0.09
(LC_xp_) BaP (ng/m^3^)	0.79	0.89	0.92	0.84	1.00	−0.02	0.19	−0.02	0.08	−0.04	−0.07	−0.07
Sex	−0.02	−0.01	0.00	0.00	−0.02	1.00	0.00	0.05	−0.05	0.19	0.09	0.72
Age	0.07	0.31	0.31	0.31	0.19	0.00	1.00	−0.04	0.21	−0.25	−0.14	−0.24
PA (min/week)	0.00	0.00	0.00	0.00	−0.02	0.05	−0.04	1.00	0.06	−0.23	−0.18	0.24
Education	0.03	0.04	0.07	0.05	0.08	−0.05	0.21	0.06	1.00	−0.13	−0.13	0.01
Eating (WHO rec)	−0.06	−0.10	−0.09	−0.07	−0.04	0.19	−0.25	−0.23	−0.13	1.00	0.63	0.07
Eating (h/unh)	−0.06	−0.07	−0.09	−0.06	−0.07	0.09	−0.14	−0.18	−0.13	0.63	1.00	0.00
V̇O_2peak_ (mL/kg/min)	−0.03	−0.09	−0.09	−0.09	−0.07	0.72	−0.24	0.24	0.01	0.07	0.00	1.00

Abbreviations: Eating (WHO rec), eating WHO dietary recommendation; LC_xp_, lifetime exposure.
